# Field-Induced Assembly and Propulsion of Colloids

**DOI:** 10.1021/acs.langmuir.1c02581

**Published:** 2022-03-03

**Authors:** Ahmed Al Harraq, Brishty Deb Choudhury, Bhuvnesh Bharti

**Affiliations:** Cain Department of Chemical Engineering, Louisiana State University, Baton Rouge, Louisiana 70803, United States

## Abstract

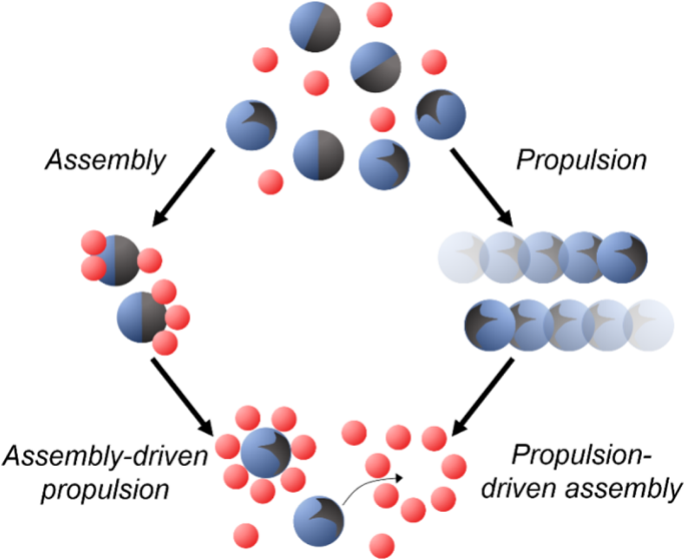

Electric and magnetic fields have enabled both technological applications and fundamental discoveries in the areas of bottom-up material synthesis, dynamic phase transitions, and biophysics of living matter. Electric and magnetic fields are versatile external sources of energy that power the assembly and self-propulsion of colloidal particles. In this Invited Feature Article, we classify the mechanisms by which external fields impact the structure and dynamics in colloidal dispersions and augment their nonequilibrium behavior. The paper is purposely intended to highlight the similarities between electrically and magnetically actuated phenomena, providing a brief treatment of the origin of the two fields to understand the intrinsic analogies and differences. We survey the progress made in the static and dynamic assembly of colloids and the self-propulsion of active particles. Recent reports of assembly-driven propulsion and propulsion-driven assembly have blurred the conceptual boundaries and suggest an evolution in the research of nonequilibrium colloidal materials. We highlight the emergence of colloids powered by external fields as model systems to understand living matter and provide a perspective on future challenges in the area of field-induced colloidal phenomena.

## Introduction

The control of colloids away from equilibrium is a fundamental challenge which may prove critical in developing future materials as well as understanding the elusive link between artificial and living objects. Widespread knowledge of colloids in static equilibrium is the reason for their ubiquity in everyday products and their use as model systems in studying the phase behavior of soft condensed matter. Recently, interest has shifted toward dynamic, out-of-equilibrium processes because of the potential in pushing the boundaries of materials design based on the properties of the colloidal building blocks. Both academics and technologists are involved in the hunt for the principles to program autonomous assembly and propulsion to actuate nonequilibrium processes. Nature builds, functionalizes, and animates objects at all scales, with unparalleled efficiency and versatility. It does so by relying not solely on elemental variety but also on mechanisms that dissipate external energy to move parts and assemble them into living matter.^1−4^ In the lab, this is recreated using colloidal particles that can consume external electric and magnetic energy to perform analogous functions.

The versatility of electric and magnetic fields is due to the ability to power nonequilibrium dissipative phenomena which can be defined as driven or active.^5,6^ The definitions of these terms have evolved in the literature^1,7,8^ and are critical to this review; they are summarized below:

(a) *dissipative*: operations that consume external energy to transition a system from an initial thermodynamic state to new states that can be in dynamic nonequilibrium or in a static kinetic trap
(b) *driven*: type of dissipative colloidal mechanism in which the structuring and motion of particles are directed by the global energy gradient
(c) *active*: type of dissipative colloidal mechanism in which the structuring and motion are governed by energy gradients local to the particles
(d) *static assembly*: assembly mechanism in which the final structure is maintained irrespective of the external energy source
(e) *dynamic assembly*: assembly mechanism forming structures that rely on transient field characteristics such as strength and frequency
(f) *passive motion*: migration of particles across a global gradient in field

**Figure 1:**
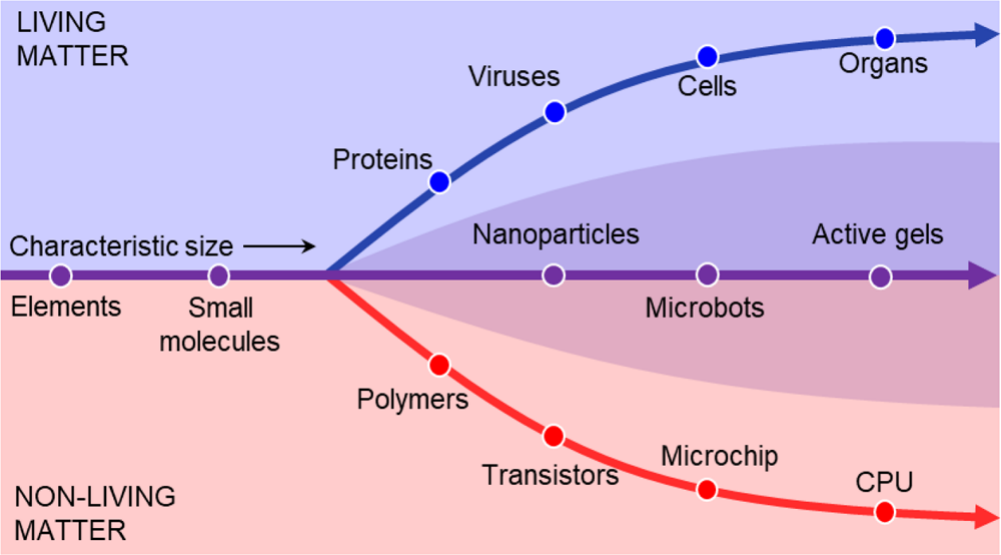
An overarching goal of studying field-induced colloidal phenomena is to understand the difference between natural and man-made objects. The divergence of living and nonliving matter is bridged by field-driven and active matter.

One of the most useful conceptual analogies of external field-induced colloidal phenomena is that of living systems, which have evolved to use biochemical energy sources for the processes of life. External fields act as the energy source for synthetic particles to perform tasks that potentially resemble life. Electrical impulses and magnetic domains provide a set of preprogrammable interactions that are tunable in small spaces and short times. Thus, colloids that would otherwise reach some thermodynamic equilibrium are instead compelled to form structures and move in their environment in a nonequilibrium fashion. The interactions induced by electric and magnetic fields play a crucial role in endowing colloids with “lifelike” features that bridge the gap between natural and man-made materials (Figure 1). Living properties originate from the ability to actively feel the surrounding environment and react to physical and chemical changes in it. In this paradigm, two phenomena stand out: colloidal assembly and propulsion in analogy, respectively, to biological self-organization and swimming.

Research in colloidal assembly and propulsion has greatly benefited from the recent progress in the synthesis and fabrication of particles with reduced symmetry.^9−17^ As will be further discussed below, the use of external electric and magnetic fields often requires the presence of an electrically conductive or magnetic domain.^18^ Depositing thin patches of metal on the surface of particles is one of the most common ways for experimentalists to introduce such domains in colloidal systems.^19,20^ These patchy particles are most often polymer or silica microspheres with thin layers (∼10^1^ nm) of metal such as gold, iron, or nickel deposited on selected areas to form patches on the surface. Many configurations can be obtained using methods such as glancing angle vapor deposition,^19^ with the most common being the Janus particle first proposed by de Gennes.^21^ This is a particle with a patch covering half of its surface resulting in two hemispheres having different physical and/or chemical properties, reminiscent of the two-faced god of Roman mythology from which it borrows the name.

**Figure 2:**
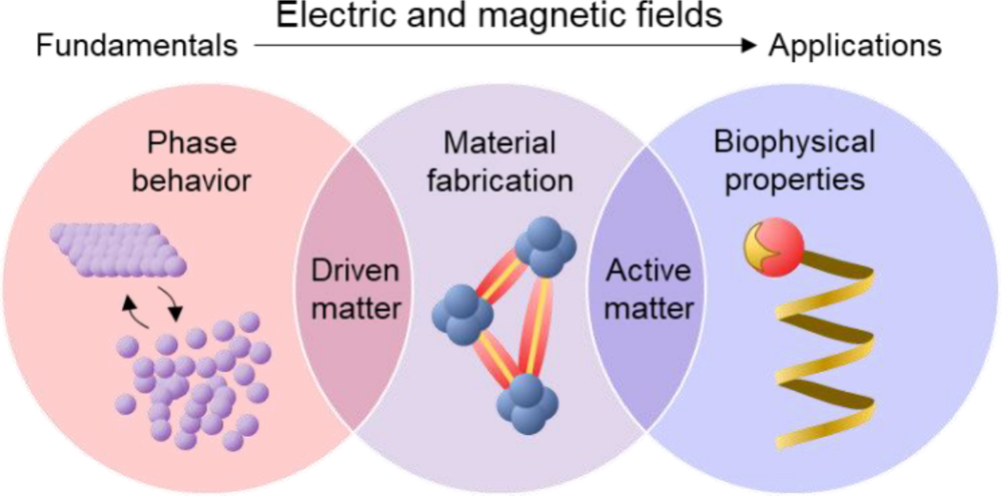
Conceptual pillars of the Invited Feature Article. The application of electric and magnetic fields on colloidal suspensions enables unique studies of phase behavior (left) that forms the basis of novel materials fabrication (center) with biophysical properties inspired by living matter (right).

Exposing colloids to electric and magnetic fields was once considered a loose intersection between the study of particle suspensions and electromagnetism. It has now acquired its own standing within the research area of soft matter. This Article aims to highlight both the unique features of using these external fields to assemble and propel colloids as well as the variety of outcomes that ensue. The principle underlined throughout the Article is the role of electric and magnetic fields in tuning the energy landscape of particles. A holistic view of the research in the field of electrically and magnetically powered soft matter is provided in Figure 2, as an intersection of material fabrication, phase transformation, and biophysical principles of colloids. The Article begins with a description of the origins of the response of colloids to electric and magnetic fields to simultaneously reveal their fundamental difference and deep similarities. The following sections classify the literature of nonequilibrium colloidal phenomena into two categories: assembly and propulsion. The most recent progress in basic science and applications is presented with the conceptual distinction of static vs dynamic assembly and passive vs active motion. We further discuss recent studies where the sharp distinction between field-induced assembly and propulsion is blurred, with descriptions of assembly-driven propulsion and propulsion-driven assembly. We conclude the Article with a perspective on the current status of research, providing summary thoughts and listing issues that require the most attention and work in the immediate future.

## Origin of the Colloidal Response to Electric and Magnetic Fields

Electric and magnetic fields morph the interaction energy landscape of particles that are susceptible to polarization. They are described by vector fields as the magnitude and direction of both electric and magnetic interactions vary in space. At colloidal length-scales, electric and magnetic fields are analogous in their effect of “polarizing” (or “magnetizing” specifically for magnetic forces) objects, i.e., inducing the formation of an effective dipole within particles. Such electric and magnetic dipoles interact in a highly directional fashion that is simultaneously attractive and repulsive, depending on the relative position of the particles and the field vector. The two fields are inherently coupled to give rise to the electromagnetic force where the electric and magnetic components originate, respectively, from stationary and moving charges. In the context of colloid science, the electric and magnetic interactions have fundamentally different origins with some consequences that affect their application in controlling colloidal assembly and propulsion.

**Figure 3:**
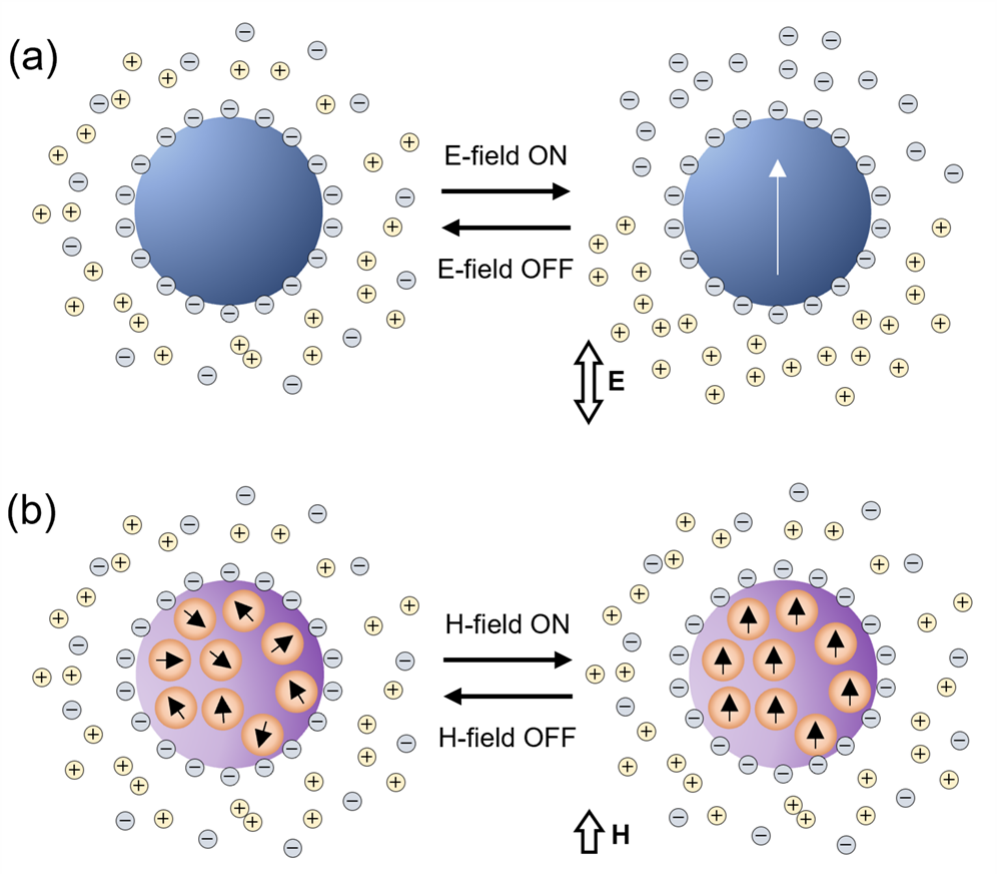
Origin of the polarization of colloids in electric and magnetic fields. (a) Applying an external ac electric field (E-field) disrupts the electrical double layer surrounding a dielectric colloidal particle in water. When the electric field is on, ions in the diffuse layer separate toward a net positive and a net negative hemisphere. (b) A colloidal particle is made paramagnetic by the presence of magnetic nanoparticles contained throughout its volume. These magnetic domains align parallel to an external magnetic field (H-field) such that the particle may be approximated to a single magnetic dipole.

In the case of electric fields, one must consider how charged colloids in water are electrically neutralized by a cloud of counterions which exist in the so-called electrical double layer surrounding the interface. The ions in the inner layer of fluid, i.e., the Stern layer, are strongly attracted to the particle and do not move.^22^ On the other hand, the ions in the outer layer, i.e., the diffuse layer, are dislocated when subjected to an external alternating current (ac) electric field (Figure 3a).^23^ Such a change in ionic cloud dislocation combines with the polarization of the core particle to form an effective electric dipole. There are also alternative cases in which the core particle is highly conductive, thus dominating the polarization in an ac external electric field irrespective of double layer charging.

Conversely, colloidal magnetic dipoles arise from the organization of atomic moments within collective domains that are broadly classified into three categories: ferromagnetic, paramagnetic, and diamagnetic.^24^ Briefly, ferromagnetic materials, e.g., iron, nickel, and cobalt, are subdivisible in domains containing permanently aligned atomic moments, yet the orientation of each domain is random in the demagnetized state and aligned with the external field when magnetized. Paramagnetic materials differ as their individual moments do not showcase any long-range order, yet they all align with an external field. Diamagnetic materials are analogous to their paramagnetic counterparts with the difference that their moments and domains align antiparallel to the external field. The effective magnetic dipole of a colloidal particle originates from the net distribution of moments that results from the alignment of local embedded domains (Figure 3b).^25^

The differences in the origins of electric and magnetic interactions are important to understand their practical advantages and limitations in driving colloidal interactions. Electric fields are effective with a large variety of particles, as the main condition for their applicability is a moderate contrast between dielectric permittivities of the particles’ counterion cloud and the solvent. This means that weakly or nonconductive colloidal particles can be easily polarized when suspended in water. Note that the chemical environment of the suspension also interacts with the electric field which both affects and is affected by the chemical species present, in particular by free ions.

Magnetic fields differ in their applicability from electric fields. They are applied in a contactless way and are chemically nonintrusive, especially at the field strengths required to manipulate colloids. The main limitation in using external magnetic fields is the range of materials that can be controlled. These must be magnetic or contain a magnetic domain embedded in them to be susceptible to the magnetic field.^6^ The instantaneous and reversible introduction of energy is a major element of the technological appeal for electric and magnetic fields. Their application offers functions that are associated with both the potential energy of interacting particles (typical of uniform fields such as gravitational) and the kinetic energy of moving particles (typical of gradient forces such as temperature and chemical). This versatility finds use in interchangeably exploiting external energy for assembling and microstructuring devices, or moving objects in complex trajectories, with inherent advantages for biomedical and microrobotic applications.

### Dipolar Intreactions in Colloids

Despite the difference in their origin, electric and magnetic field-induced effects in colloidal assembly share a conceptual background; i.e., they can both be analyzed using the point-dipolar approximation. Through this theoretical tool, a nano- or microparticle polarized by an external field is imparted with a net dipole moment. This approximation is often useful to predict and interpret both electric and magnetic field-induced phenomena. A field-exposed particle may be approximated as an electric point-dipole, ***p***_e_, or a magnetic point-dipole, ***p***_m_, expressed by^26^
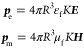
1where *R* is the radius of the particle, and ε_f_ and μ_f_ are, respectively, the electrical permittivity and the magnetic permeability of the medium. ***E*** and ***H*** are the vectors of the electric and magnetic fields, respectively. Equation 1 highlights the scaling of the moments with the particle volume, which is an underlying limitation when it comes to polarizing sub-nanometer domains. The term *K* represents the real part of the Clausius–Mossotti function which provides a measure of the degree of polarization of a particle. This is based on a contrast of electric permittivity or magnetic permeability between the particle i and the surrounding fluid, f:^26^
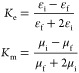
2Here, the subscripts e and m refer to electric and magnetic versions of the equation, respectively. The Clausius–Mossotti function reveals a critical feature of dipolar interactions: the effective polarizability of a particle depends on the difference in permittivity/permeability of colloids and the suspending medium and is not solely intrinsic to the material in isolation.^27^ For example, when μ_i_ > μ_f_, the effective magnetic moment of a particle has a positive sign, and the particle behaves paramagnetically; i.e., its dipole aligns parallel to the external field. By contrast, when μ_i_ < μ_f_, ***p***_m_ becomes negative leading the particle to behave diamagnetically, i.e., with its dipole aligned antiparallel to the applied field. This latter case is the fundamental concept underlying “negative magnetophoresis”^28^ and the manipulation of nonmagnetic particles in magnetic fluids. The analogous phenomenon of “negative dielectrophoresis” exists for electric field manipulation.^29^

When the applied electric or magnetic field is uniform, the moment of a particle i communicates with the moment of a second particle j at a distance σ through a long-range potential that gives rise to the interaction energy *U*:^30,31^
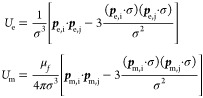
3Equation 3 shows the scaling of point-dipolar interaction energy with the square of the external field strength ***E*** and ***H*** appearing, respectively, in ***p***_e_ and ***p***_m_. Note that the above equations are applicable to colloids in a state where σ ≫ *R*. Point-dipole approximations are a commonly used tool to understand electromagnetic interactions in colloids. These relatively straightforward calculations are particularly useful in predicting pseudoequilibrium structures observed after exposing particles in suspension to external electric and magnetic fields.

### Field-Induced Propulsion Mechanisms in Colloids

While electric and magnetic field-induced assemblies share an analogous point-dipolar approximation, the analytical treatment of active propulsion differs quite significantly between the electrically and magnetically powered cases. Both fields generate propulsion by inducing asymmetric forces on the swimming objects because of asymmetry in shape and/or surface. The difference is that the effects of the electric field extend to the suspending medium with most active motion deriving from an imbalanced electro-osmosis on the surface of an anisotropic particle. Conversely, magnetic fields only act on the magnetic domain of the suspension, i.e., the ferromagnetic active particle and not the medium. In this case, the motion is achieved by nonreciprocal reorientation of the anisotropic particle by the magnetic torque which causes a drag imbalance on the surrounding fluid.

Induced-charge electrophoresis (ICEP)^32−34^ is a successful analytical tool to describe active motion in ac electric fields which will be discussed in further depth below. Briefly, the phenomenon indicates the motion of a metallodielectric particle propelled because of asymmetric fluid flow caused by asymmetric charging of the particle. At low field strengths and frequencies, the translational and rotational velocity, *V* and Ω, respectively, of the active particle propelled by ICEP is given by the following:^32,34^
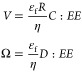
4where η is the viscosity of the solution, and *C* and *D* are dimensionless tensors that share the symmetry of the particle and depend on its shape and composition. Due to the role of the electric field in polarizing the counterion layer around the particle, ICEP is conceptually closer to fully locally driven active matter such as catalytic Janus particles. While powered by an external electric field, the role of the global uniform field (time-averaged) is to induce a local field gradient in the vicinity of the geometric boundaries of the particle.

That is not the case for magnetically actuated active motion, in which the external field magnetizes the particle which swims because of hydrodynamic coupling. Therefore, no equivalent of ICEP currently exists for the case of magnetic active propulsion. Instead, several successful models exist that analyze either the individual swimming of a particle with a specific geometry^35^ or the collective motion of flocking ferromagnetic rollers.^36^

## Field-Induced Assembly of Colloids

Colloidal assembly is the arrangement of micro- and nanoscale particles into structures of well-defined symmetry and configuration. The individual components involved in the assembly process are building blocks connecting to form so-called *suprastructures*, in an analogy between the fields of supramolecular synthesis and supracolloidal assembly.^37^ The defining characteristic of assembly is its bottom-up nature, for which the final architecture is the net effect of the interactions among the building blocks and their packing. External electric and magnetic fields provide an additional interaction with a set of controllable parameters, namely, the strength, frequency, and orientation of the field, which act together with preexisting interactions to drive assembly. Colloidal particles normally interact via nonspecific interactions such as van der Waals attraction and electrostatic repulsion and follow a thermodynamic pathway toward an equilibrium configuration.^22^ The exposure of polarizable particles to external fields implies the redrawing of their interaction energy landscape.^38^ This is how external forces direct assembly, effectively “pushing” particles away from equilibrium toward an alternative thermodynamic pathway, which is not accessible without the assistance of the field. Thus, in all assembly mechanisms that are driven by electric and magnetic fields, the dissipation of external energy pays the cost of veering off the thermodynamic pathway. Research in field-induced assembly is broadly classified into two categories equivalent to two types of dissipative assembly.^1^ On one hand, there are assemblies of static suprastructures which maintain their ordered configuration once the external field is removed.^39,40^ On the other hand, there are assemblies of dynamic suprastructures, which only exist while external energy is supplied and will disassemble into the constituent building blocks when the field is removed.^3^

**Figure 4:**
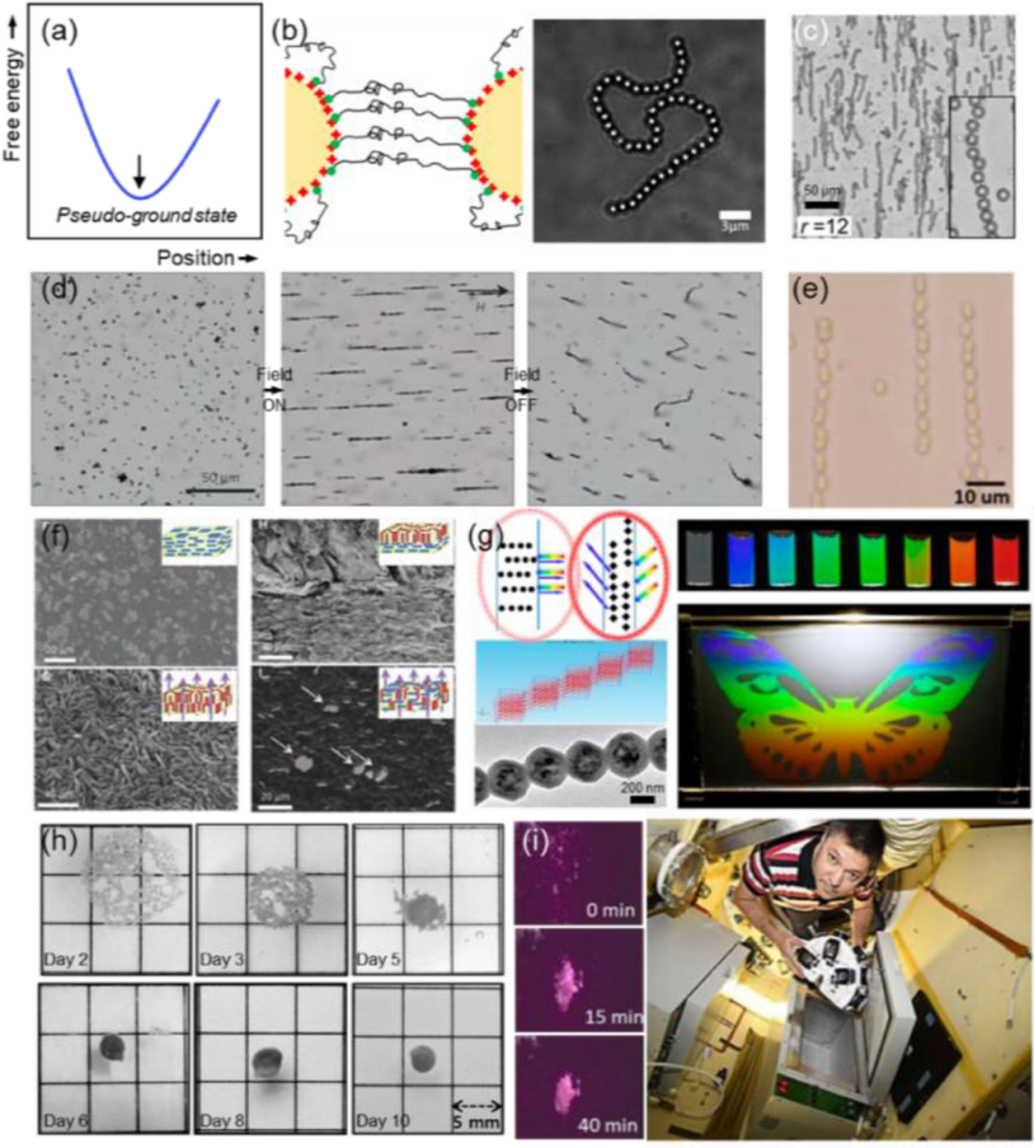
Driven assembly of static structures. (a) Particles in an external electric and magnetic field assemble into a pseudoground state, in which they can be trapped by introducing other physicochemical interactions. (b) Schematic (left) of DNA linkages between colloidal particles that are assembled into chains using a magnetic field. Once the field is removed, the chains maintain their structure as shown in the micrograph on the right. Adapted with permission from ref (46). Copyright 2014 American Chemical Society. (c) Chains assembled from oppositely charged polystyrene microspheres. The large particles (4 μm diameter) have a negative surface charge while the small particles (0.9 μm diameter) have a positive surface charge. The chains are formed by the application of an ac electric field and remain permanently bound by electrostatic interactions. Adapted with permission from ref (45). Copyright 2014 American Chemical Society. (d) Nanoscale capillarity binds iron oxide nanoparticles that are coated with fatty acid. Magnetically assembled chains of such nanoparticles maintain their shape upon removal of the external field. Adapted with permission from ref (47). Copyright 2015 Springer Nature. (e) Chain of Synechococcus PCC7002 cyanobacteria assembled via an ac electric field onto a flexible polyelectrolyte substrate. The cells maintain their assembled structure and pattern the substrate, while preserving their photosynthetic pigment integrity. Adapted with permission from ref (48). Copyright 2017 American Chemical Society. (f) Polyurethane-based composites are microstructured using magnetic fields to align alumina platelets covered in iron oxide nanoparticles. The material reinforcement enhances its tensile strength, wear resistance, and flexural modulus based on the magnetic alignment of the platelets. Adapted with permission from ref (49). Copyright 2012 American Association for the Advancement of Science. (g) Edge-to-edge magnetic assembly of iron oxide nanocubes, encapsulated in a layer of silica. The material shows an orientation-dependent photonic response. Adapted with permission from ref (51). Copyright 2019 American Chemical Society. (h) Fibroblast cells (BALB 3T3 cell line) containing magnetic microparticles and assembling into a disk and subsequently into a spheroid within 10 days. Adapted with permission from ref (56). Copyright 2014 Wiley Periodicals. (i) Chondrosphere spheroid fusion in a gadolinium complex under a controlled static field inside the International Space Station. A lower salt concentration ensures the cell viability of the medium. Adapted with permission from ref (57). Copyright 2020 American Association for the Advancement of Science.

Static assembly mechanisms take advantage of the external fields to access specific arrangements of the building blocks and include further interactions and/or processing to ensure their permanent binding (Figure 4a).^41,42^ By tuning the relative alignment of the field and the particle suspensions, it is possible to control the directionality of the suprastructures. For example, one-dimensional assembly under electric and magnetic fields is manifested in the formation of chainlike structures.^43−45^ These form as particles that acquire dipoles oriented in the same direction, thus attracting each other in parallel with the field and repelling each other when orthogonal to the field (Figure 4b–e). Byrom et al. have demonstrated the magnetic assembly of chains with controlled flexibility, using DNA linkers to bind particles and maintaining the structure after removing the magnetic field, as shown in Figure 4b.^46^ A similar interlinking of particles can be achieved by using a pair of oppositely charged particles of dissimilar sizes in an external ac electric field (Figure 4c).^45^ Iron oxide nanoparticles coated with fatty acid can be magnetically assembled into ultraflexible chains that are bound by nanocapillary bridges (Figure 4d).^47^ Similar principles also allow the patterning of substrates with cells (Figure 4e).^48^

The main role of the external field in such processes is to provide order and directionality to the structure that would not otherwise form. Such uniform, static fields find functional applications in many material assemblies. Composites can be built with reinforcing elements coated with superparamagnetic nanoparticles (Figure 4f).^49^ This controls the distribution and orientation of these elements where a magnetic field determines the properties of the material such as stiffness, wear resistance, and the shape memory effect. Photonic crystals are assembled very efficiently using external fields, achieving a variety of structural colors^50^ as well as orientation-dependent properties (Figure 4g).^51^ Billaud et al. structured the surface of a graphite electrode using an external magnetic field.^52^

One major area of applications of external fields is in the control of biological matter. This includes the manipulation of cells, subcellular aggregates, and even biomolecules under the influence of electric and magnetic fields (Figure 4e,h,i).^53−55^ 3D bioprinting is a prime example which has seen progress in recent years through the development of techniques for tissue engineering using magnetic fields. This method is investigated as an alternative to conventional cell culture and tissue fabrication strategies involving the use of scaffolds. The magnetic field replaces the scaffold and provides the necessary force to levitate cells and spheroids, allowing them to fuse into a single bioassembled structure (Figure 4h).^56^ Originally, this required the introduction of a magnetic domain in the cell in the form of iron oxide nanoparticles often coated to prevent cytotoxicity. Alternatively, the culture takes place in a paramagnetic salt solution allowing the manipulation of cells as diamagnetic objects, according to the concept of *negative magnetophoresis* discussed above. The main drawback is that levitating heavy objects requires higher concentrations of paramagnetic ions, often gadolinium (Gd^3+^) chelates, which surpass the toxicity limit of cells. Parfenov et al. bypassed the issue by performing a bioassembly aboard the International Space Station where gravity does not contribute to the force balance on cells (Figure 4i).^57^ They dispersed human chondrocytes in a 10 mM gadobutrol solution, a Gd^3+^-based medium at a concentration lower than the toxicity limit. In the absence of gravity, the low concentration of gadobutrol is sufficient to allow assembly and fusion of chondrospheres into a tissue without affecting cell viability.

**Figure 5:**
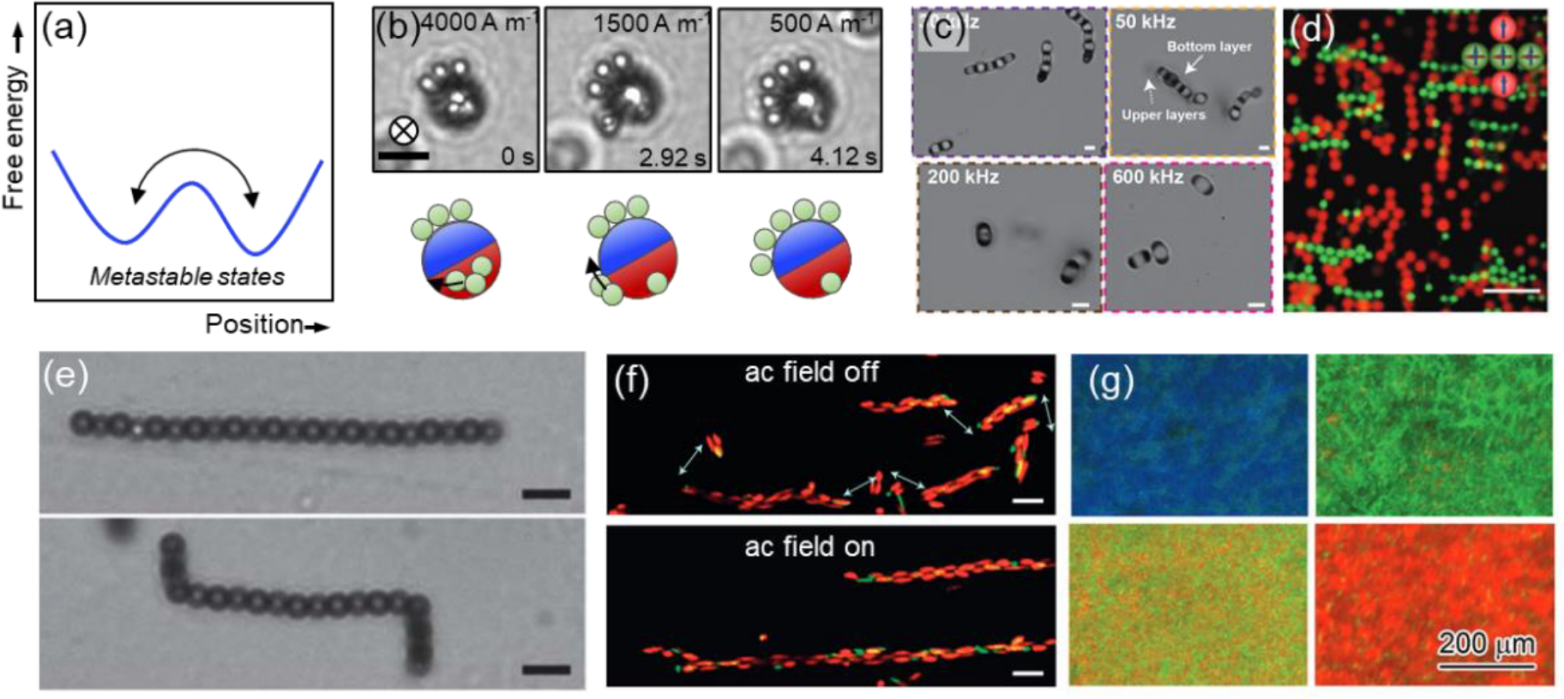
(a) Dynamic assembly depends on a transient energy landscape. Particles arrange into reversible configurations associated with metastable states. (b) Isomerization of a colloidal supraparticle involving the relocation of “satellite” microparticles between hemispheres of a “core” Janus particle. As the magnetic field intensity is lowered, satellites migrate from the polymer hemisphere on top of the core particle toward the metal patch on the side. Scale bar: 5 μm. Adapted with permission from ref (9). Copyright 2020 American Association for the Advancement of Science. (c) Based on the frequency of the ac electric field, particles with two gold patches assemble into various suprastructures: (top left) alternating chain; (top right) open-brick wall; (bottom left) staggering out-of-plane chain; and (bottom right) T-shape. Scale bar: 2 μm. Adapted with permission from ref (65). Copyright 2021 American Chemical Society. (d) Paramagnetic (green) and nonmagnetic (red) polystyrene microspheres in orthogonal electric and magnetic fields. The magnetic particles assemble into chains aligned with the external magnetic field while the red ones assemble in the normal direction, aligning with the electric field. Scale bar: 20 μm. Adapted with permission from ref (66). Copyright 2016 Royal Society of Chemistry. (e) Iron oxide-capped Janus particles in orthogonal electric and magnetic fields. Particles form a mixed staggered and double chain after a few seconds of field application. Scale bar: 5 μm. Adapted with permission from ref (67). Copyright 2013 Royal Society of Chemistry. (f) Fibrillar microstructures self-assembled from gold-capped Janus ellipsoids. An application of an ac electric field promotes the elongation and contraction of the structures. Scale bar: 5 μm. Adapted with permission from ref (69). Copyright 2015 Springer Nature. (g) Color microscope images of photonic crystals of cerium oxide particles assembled via an ac electric field. As the field strength is lowered from 3.5 to 0 V, the color changes from blue to green, yellow, and red. Adapted with permission from ref (74). Copyright 2018 Wiley-VCH.

Dynamic assembly occurs when the interactions responsible for the formation of a specific suprastructure vary in time. External fields are suited for this type of assembly because they provide control in four dimensions: the three spatial coordinates plus time.^58^ This is achieved by controlling the current input to program the magnitude and frequency of the fields. Such regulation of the energy input underlies the concept of a dynamic energy landscape, in which particles reversibly switch from polarized to nonpolarized.^59^ The result is the formation of two or more transient suprastructures corresponding to different metastable configurations that depend on the characteristics of the external field (Figure 5a).^60−63^ The two primary states of dynamic assembly correspond to the on and off states of the electric and magnetic fields.^64^ Generally, these are particle systems that exist in a random state when the field is off and subsequently acquire an assembled order when the field is on. An assembly of supraparticles was recently reported from a dispersion of microparticles, some of which were coated with a 30 nm thick iron patch.^9^ When exposed to an external magnetic field, patchy particles attract the isotropic “satellites” to form clusters of a controlled configuration. Reducing the field intensity leads to the reconfiguration of some of the clusters in which satellites travel from one location to another of the same core patchy particle (Figure 5b). This is an example of a dynamic energy landscape allowing particles to switch between more than 2 states. Wang et al. reported the formation of suprastructures that transition from a chainlike to an open-brick wall configuration (Figure 5c).^65^ To obtain this, they synthesized particles with two gold patches and directed their assembly using an ac electric field. Upon crossing a frequency of 50 kHz, the chain assemblies develop in the orthogonal direction to form the open-brick wall. Multiple external fields can also be applied simultaneously to form higher-order structures which depend on both the spatial and temporal configuration of the fields (Figure 5d,e).^66−68^

Dynamic assembly points to a new concept of material which has properties and functions that depend on the state of its constituent building blocks as directed by the user. An example of this was showcased by Shah et al. with the assembly and ac electric field-induced actuation of chains made of ellipsoidal Janus particles.^69^ These are elongated polystyrene microparticles that are half coated with a 15 nm thick gold layer. The particles self-assemble through van der Waals and electrostatic interactions, equilibrating into fibrillar microstructures. When exposed to the external electric field, these structures rapidly and reversibly elongate and contract (Figure 5f).^69^ The main application of dynamic field-induced assembly is the transformation of electrical input into mechanical energy. Both magnetic and electric fields are used as modulators of suspension rheology, by inducing the assembly of particles in magneto- and electrorheological fluids.^70^ Based on the intensity of the applied field, these fluids have variable moduli and can transition from Newtonian to Bingham fluids. These can find use in the active control of many mechanical devices such as valves and clutches but also in biomedical applications including artificial joints. When coupled with sensors, these fluids become so-called smart materials that respond to external variations to perform different functions.^71^ The fabrication of tunable photonic crystals is another area of technological interest for dynamic assembly.^72^ Traditional methods often involve processing steps to separate the solid crystalline material. Conversely, the material can be maintained in solution and actuated dynamically using external fields.^73^ By changing the strength of the electric field, Fu et al. reversibly compressed and decompressed a lattice of silica-coated cerium dioxide nanoparticles in propylene carbonate. The particles and medium have a large dielectric constant, allowing for a wide photonic band gap with saturated colors that change based on the strength of the external field (Figure 5g).^74^

**Figure 6:**
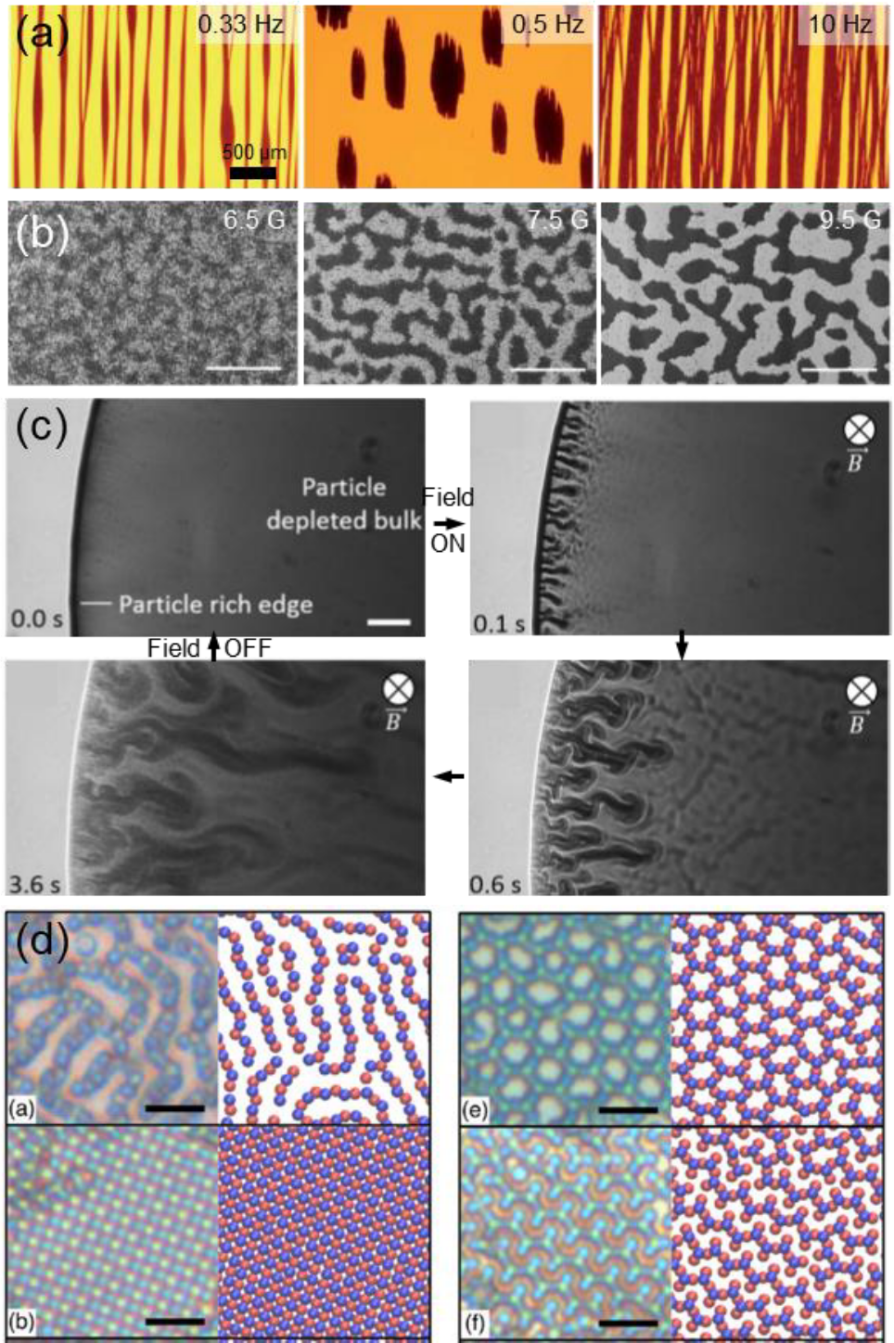
(a) Magnetic field-driven assembly of percolated or condensed regions of paramagnetic particles at a constant field strength (1500 A m^–1^) and different frequencies. Adapted with permission from ref (78). Copyright 2014 Royal Society of Chemistry. (b) Phase separation of paramagnetic particles after exposure to high-frequency rotating magnetic fields of different strengths. Scale bar: 100 μm. Adapted with permission from ref (79). Copyright 2020 Royal Society of Chemistry. (c) Edge of a drying droplet containing iron oxide nanoparticles. Applying an out-of-plane magnetic field triggers magnetostatic microconvection that disappears upon removal of the field. Scale bar: 50 μm. Adapted with permission from ref (80). Copyright 2018 American Chemical Society. (d) ac electric field-driven assembly of polystyrene microparticles. The particles are semiconfined in two layers using an additional dc electric field out-of-plane. Changing the field strength, they formed a wormlike phase (top left), a honeycomb-Kagome lattice (top right), a square bilayer (bottom left), and a zigzag stripe lattice (bottom right). Adapted with permission from ref (81). Copyright 2021 American Chemical Society.

The arrangement of colloidal particles is a powerful tool to observe and characterize basic physical phenomena involving atoms and molecules. The analogy of colloids as big atoms^75,76^ stems from the similar Brownian motion intrinsic to units of matter that are small enough to be susceptible to thermal noise. Colloids, unlike atoms, can be easily observed with a microscope and were used by Perrin as experimental proof of Einstein’s theory on the atomic nature of reality.^77^ Since then, colloidal assembly and disassembly have been used to reveal such fundamental dynamics as crystal nucleation, phase transitions, and glassy arrest. In this context, electric and magnetic fields control the distribution of energy in space and time, across a colloidal suspension. By controllably morphing the energy landscape, external fields augment the range of dynamics accessible by spontaneous assembly. For example, Swan et al. described the correlation between the frequency of an external magnetic field and the resulting state of particles.^78^ Toggling the magnetic field at a frequency lower than the relaxation rate of the suspension allows rearrangement of particles. Conversely, higher frequencies trap structures in kinetically arrested states. Paramagnetic particles arrange themselves in percolated chainlike structures or crystalline clusters depending on the interplay between their structural relaxation time and the frequency of toggling (Figure 6a,b).^79^ Phase behavior is also heavily dependent on the geometric confinement of the constituent building blocks. A natural geometric boundary is found in drying droplets where the degree of confinement increases from the center of the droplet to the pinned edge. Due to the spontaneous transport of the particles during the drying process, a magnetic nanoparticle-rich confined state is generated at the droplet edge. The asymmetric distribution of the nanoparticles leads to the generation of a magnetostatic convection from the edge to the center upon the application of a magnetic field as shown in Figure 6c.^80^ Electric fields have recently been used to simultaneously control the confinement of microparticles and the strength of their dipolar interactions. Maestas et al. employed a direct current (dc) electric field to arrange particles into two separate layers at the two electrodes. To this, they coupled an ac field used to control the magnitude of the dipolar interaction strength and found that the confined layers respond to the change in energy landscape by forming a variety of complex phases such as zigzag stripes, honeycomb-Kagome, and sigma lattices as well as tetramer networks (Figure 6d).^81^

## Self-Propulsion of Active Colloids

**Figure 7:**
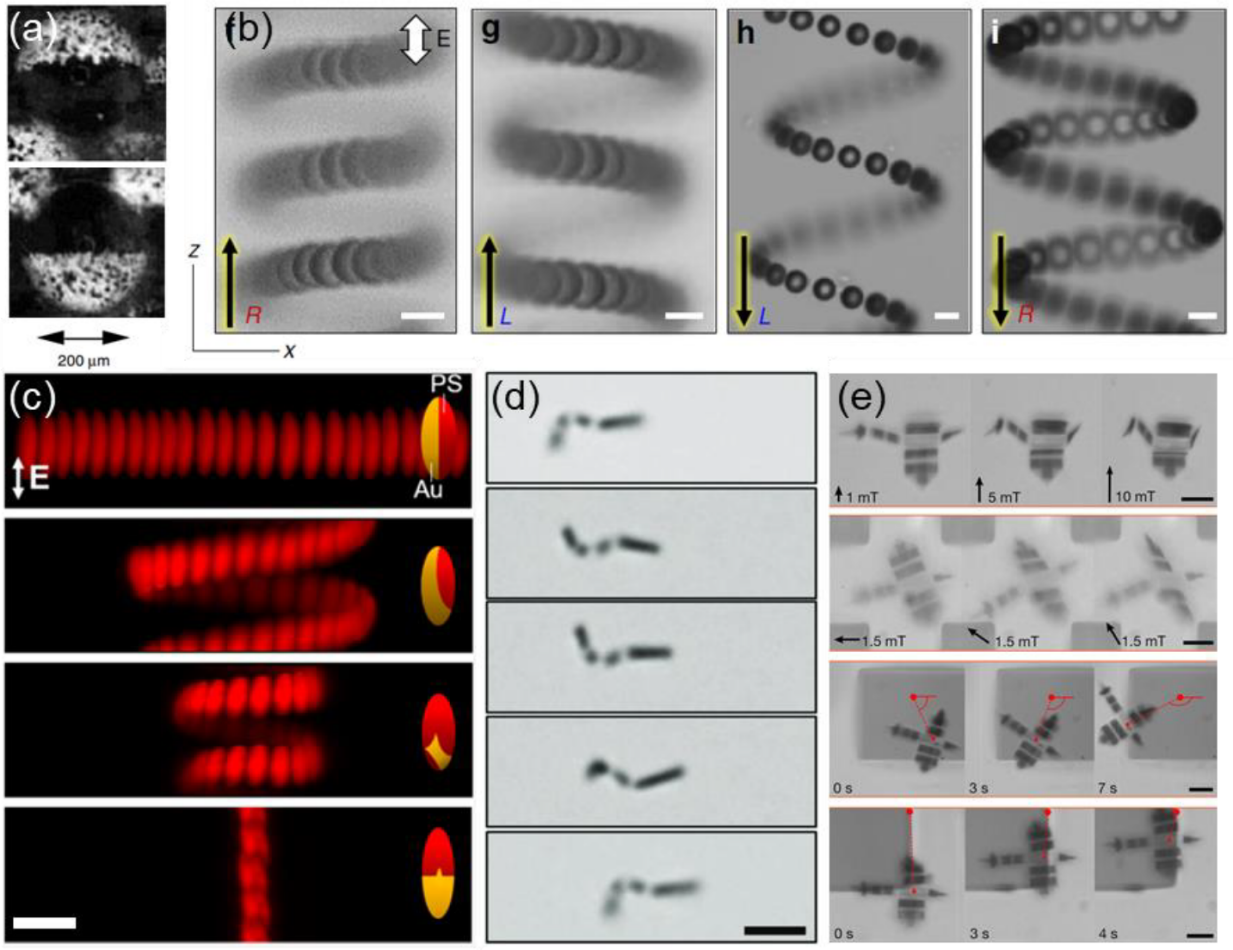
(a) Microcapsule containing negatively charged white microparticles and positively charged black microparticles. Two electrodes sandwich the capsule and allow the application of an electric field. A low-power dc field drives the separation of white and black particles, establishing the electrophoretic equivalent of a pixel. Adapted with permission from ref (84). Copyright 1998 Springer Nature. (b) Superimposed micrographs showing the ICEP propulsion in a helical trajectory of a polystyrene particle with a triangular gold patch. The direction of motion and the handedness (right, R; and left, L) of the helical trajectory depend on the initial particle orientation when the ac electric field is applied. Scale bar: 5 μm. Adapted with permission from ref (11). Copyright 2019 Springer Nature. (c) Polystyrene ellipsoids coated with gold patches of various symmetries undergoing ICEP propulsion. Coupling shape and surface anisotropy gives rise to different motions, from 1D translation to 2D circular orbiting to 3D helical trajectories. Scale bar: 5 μm. Adapted with permission from ref (10). Copyright 2021 American Chemical Society. (d) Image sequence showing one cycle of motion of a magnetically propelled nanofish. The nickel segments at the center of the swimmer continuously realign with the external field while the gold segment undulates causing the necessary fluid flow for net translation. Scale bar: 2 μm. Adapted with permission from ref (95). Copyright 2016 Wiley-VCH. (e) Microscopic “bird” constructed from magnetically patterned panels in an origami-like structure. The movement of the structure depends on the strength and frequency of the external oscillating magnetic field. Images from top to bottom show, respectively, movement of the microbird by flapping its wings, hovering, turning, and sideslipping. Scale bar: 30 μm. Adapted with permission from ref (96). Copyright 2019 Springer Nature.

External electric and magnetic fields have a profound effect on the dynamics of colloidal particles and can be used to power their motion. It is necessary to recognize that such motion generally results from the occurrence of a spatial gradient in field intensity. In fact, a polarized particle in a homogeneous external field will not move. If the particle experiences a global field gradient, it will migrate toward or away from the gradient vector depending on its value of the Clausius–Mossotti function (eq 2). Motion driven by a global electric and magnetic field gradient is referred to as electro- and magnetophoresis, respectively, and may be considered as a type of passive motion, similar to sedimentation occurring in the gravitational field.^82,83^ This is in contrast with active motion, which refers to the propulsion of colloids that occurs from the coupling of fluid flows with local field gradients.^32,33^ Such local gradients often result from asymmetry in the particle shape and/or surface properties. Field-induced phoresis is of interest in many applications ranging from lab-on-chip to the commercially available electronic inks made by e-ink (Figure 7a).^84^ The focus of research in recent years has drastically shifted toward the active motion of colloids. The transition from field-driven to truly active systems implies that the rules governing the trajectory of a colloidal particle are intrinsic to the physical and chemical configuration of its shape and surface with respect to the surrounding fluid. An active particle in an external electric and magnetic field does not simply experience the global field gradient but generates a local gradient near its surface. Coupled with fluid flows, this local energy inhomogeneity guides the motion of particles in complex trajectories that do not necessarily follow the global field gradient. Active colloids are fundamentally related to swimming microorganisms. On one hand, the natural world is a source of inspiration for the design of synthetic active particles with programmed motion. On the other hand, artificial microswimmers are ideal model systems to study the biophysical principles that govern the individual and collective motion of living organisms. This dual relationship of biological inspiration and translational discovery underlies the use of the term “living” with synthetic colloids.

It is necessary to recognize two ever-present challenges in the field of active particles. The motion of colloids is inherently randomized by Brownian diffusion due to their small size.^22^ Thus, the inherent stochasticity of colloids makes programming their motion a complex task. In addition, the fluid dynamics of microscopic objects is characterized by low Reynolds number, *Re* = *ρvL*/η, where ρ and η are the density and viscosity, respectively, of the fluid, while *v* and *L* are the velocity and characteristic length, respectively, of the swimmer.^85^ The *Re* number is a measure of the ratio of inertial to viscous forces acting on an object immersed in a fluid. Colloids have *L* ∼ 10^–6^ m such that their *Re* in water is much smaller than 1 meaning that inertial forces become negligible compared to viscous stresses. Thus, from the perspective of a colloidal microswimmer, water feels as viscous as honey to humans. Also, the consequence of negligible inertial forces is the so-called scallop theorem. This states that, at low *Re*, reciprocal strokes such as the opening and closing of a scallop do not produce net displacement because of the time-reversal symmetry of the motion.^85^ Therefore, the fabrication and propulsion of active colloids always have to contend with the challenges of stochasticity and low-*Re* fluid properties.

Despite the intrinsic obstacles, nature found a way to achieve dynamic motion at a low *Re* number and has recently inspired the design of active systems powered by external electric and magnetic fields. The basic mechanism for the active propulsion of colloids in electric fields is the ICEP of metallodielectric Janus particles.^32,33,86,87^ These are microparticles coated in one hemisphere with a conductive layer of metal, typically gold. Charges in the double layer of a particle rearrange in response to an ac electric field, acting to screen it. This induced charging causes electro-osmotic flows that, in an isotropic sphere, are symmetric along the field axis and its normal. The Janus configuration breaks the symmetry in the induced charge, as the gold-coated hemisphere is highly conductive resulting in higher electro-osmotic flow localized near the patch. The unbalanced flows caused by the local electric field gradient drive the motion of the particle in the direction perpendicular to the global field. The trajectory of motion in ICEP is governed by the overall symmetry of the active particle, which can be tuned by its shape and surface.^34,88^ For example, two Janus particles bound in a doublet have been shown to spin and rotate under an ac electric field.^89^ This occurs as each Janus particle attempts to move perpendicular to the field in the direction of the dielectric hemisphere, leading to rotation of the bound pair. Glancing angle vapor deposition allows the fabrication of patchy particles with even lower symmetry than the Janus hemispherical configuration. This technique was recently used to demonstrate the unique role of surface anisotropy in encoding complex motions under ac electric fields.^10,11^ Spherical polystyrene microspheres coated with low-symmetry patches of gold swim in nonlinear 3D trajectories. Notably, a triangular patch allows particles to move in a helical path similar to the swimming of spermatozoa and other microorganisms.^90^ Experimental evidence and analytical modeling show that this occurs because the triangular metal patches align their plane of mirror symmetry at an oblique angle with respect to the field axis. Thus, these complex patchy particles rotate and translate about the field axis to trace helical trajectories (Figure 7b).^11^ The trajectory of an ICEP-propelled particle is not only determined by the asymmetry of surface patches. This can couple with the shape anisotropy to give rise to a wide array of active particle motions. Polystyrene ellipsoids are readily obtained by stretching spherical microparticles to a specific aspect ratio.^15,83^ Such anisotropic particles can then be rendered patchy using the same metal vapor deposition techniques used for microspheres but yielding more complex patch shapes. Lee et al. reported a way to fabricate and characterize such particles based on the patch asymmetry, measured from the metal coverage of the transverse and longitudinal axis of the ellipsoid.^10^ These active particles access linear, circular, and helical motions based on the type of gold patch on their surface (Figure 7c).^10^ For the helical motion, as the longitudinal symmetry of the patch increases, the helical pitch generally decreases. Also, as the transverse symmetry increases, the helix diameter decreases. Patchy particles in ICEP motion are normally oriented with their dielectric side forward and the metal patch backward because of the higher electro-osmotic flows on the metal patch. A form of active motion also occurs with gold-coated Janus particles oriented with the patch side forward, in vertical electric fields between two indium–tin oxide (ITO)-coated substrates and at very high electric field frequency.^91^ This was termed self-dielectrophoresis (sDEP) indicating the occurrence of a localized field gradient between the gold and the ITO surface which leads to active motion of single particles. On a similar note, unbalanced electrohydrodynamic (EHD) flows on colloidal dimers showcase active propulsion with a frequency-controlled orientation.^92^

Time-varying magnetic fields work similarly, albeit with some key differences, to power the active motion of colloids. The main difference between active motion in electric and magnetic fields is that, in the former, the local field gradient extends to the fluid causing unbalanced flows. Magnetic fields generally act only on the magnetic parts of a colloidal system, generating a torque that acts to align the particle with the field axis.^93^ In a static homogeneous field, once aligned, a magnetized particle remains immobile. In a time-varying magnetic field, which originates from a rotating or oscillating ac electric field, the magnetic torque acts to continuously realign the particle.^94^ Thus, magnetic fields can induce active motion through anisotropy in particle shape and surface to overcome the scallop theorem. Li et al. fabricated flexible active nanoswimmers by joining together nanowire segments of gold and nickel with nanoporous silver joints.^95^ The fishlike structure undergoes undulatory motion when exposed to an oscillating magnetic field. The nickel sections of the nanoswimmer are placed in the center of the body, with the gold segments forming the head and the tail. This promotes the largest possible deformation of the structure from the oscillating magnetic field, which causes a propulsive wave along the axis of the nanowires (Figure 7d). Similar to the emulation of fish, the bird anatomy is another example of successful bioinspiration in the fabrication of magnetic active systems. Cui and co-workers fabricated a micromachine by connecting panels made of poly(methyl methacrylate) coated with 60 nm of cobalt.^96^ Initially, the panels are encoded with specific magnetic patterns using coercive fields between 30 and 140 mT. Following this, they are actuated by an alternating magnetic field to fold, bend, and twist the structure suspended in solution. A design of the pattern of magnetization of each panel coupled with the tuning of the field characteristics allowed the fabrication of a microscopic bird with outstanding control over its translational and rotational movement (Figure 7e).

**Figure 8:**
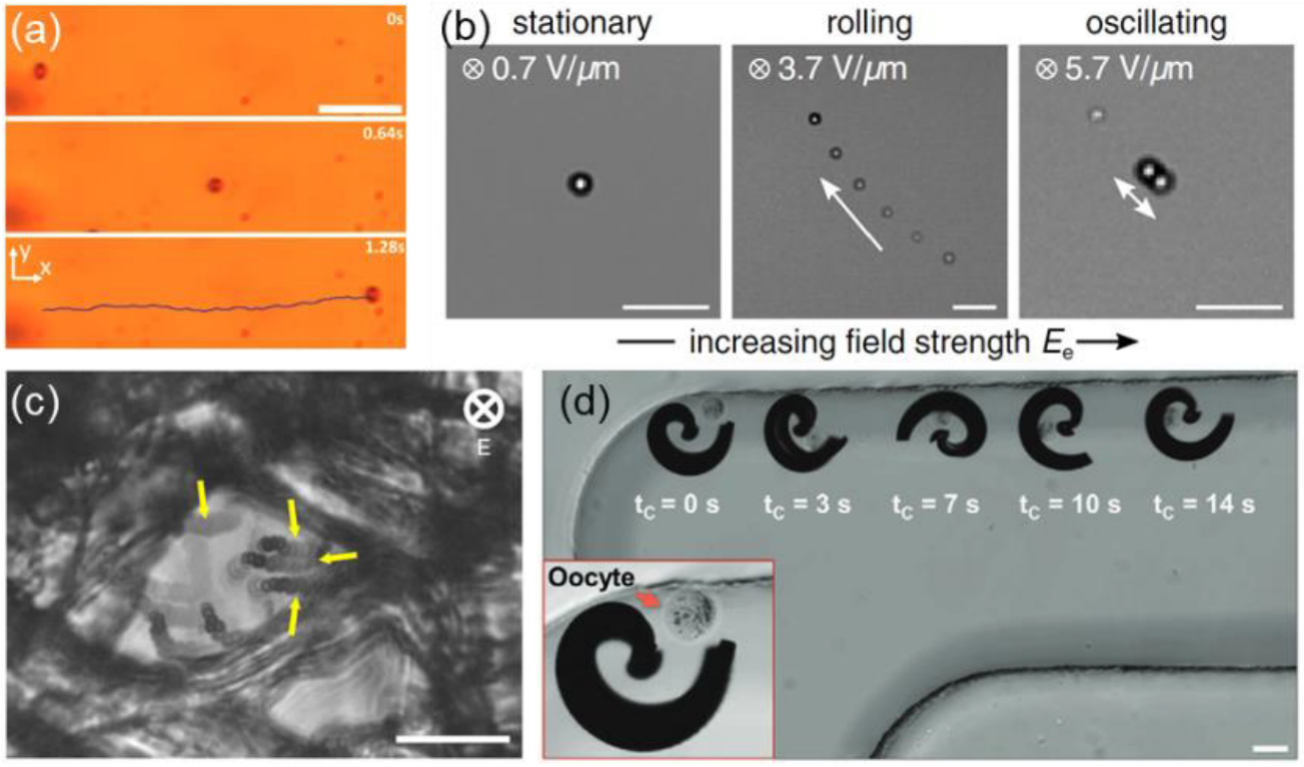
(a) Rolling motion of a hematite ellipsoid under a rotating magnetic field. Scale bar: 5 μm. Adapted with permission from ref (101). Copyright 2017 Wiley-VCH. (b) Quincke roller composed of a polystyrene microsphere in an AOT-hexadecane solution. Upon an increase in the strength of the dc electric field, the particle begins rolling and eventually enters an oscillating regime. Scale bar: 40 μm. Adapted with permission from ref (103). Copyright 2021 American Physical Society. (c) Superimposed microscope images showing active particles tunneling through a cross-linked cellulose matrix. Scale bar: 20 μm. Adapted with permission from ref (11). Copyright 2019 Springer Nature. (d) Spiral-shaped micromotor actuated by a rotating magnetic field to capture and transport a murine zygote cell. Scale bar: 100 μm. Adapted with permission from ref (105). Copyright 2020 Wiley-VCH.

So-called surface rollers or walkers are a separate class of active particles that are receiving considerable attention.^97−99^ The difference with the microswimmers described above is that rollers and walkers rely on the proximity of a substrate with which they interact.^100^ The boundary layer of fluid adjacent to the substrate has a higher apparent viscosity that causes a drag imbalance on particles, leading to their rolling or walking on the surface. This principle is well-demonstrated by Martinez-Pedrero et al. using ellipsoidal hematite colloids.^101^ These are weakly ferromagnetic particles that roll perpendicular to the long axis when exposed to a rotating field (Figure 8a). The rolling motion is highly dependent on the frequency of the magnetic field. Below a certain critical frequency, the particle displays a net motion as its rotation is hydrodynamically coupled with translation over the substrate. Above the critical frequency, the rotation of the ellipsoid falls out of synchronization with the field, and the translation is replaced by a back-and-forth motion. Equivalent rolling was recently shown with simple isotropic paramagnetic spheres immersed in a mucus fluid.^102^ Here, the symmetry breaking is associated with nonlinearities in the viscoelastic response of non-Newtonian fluids to the torque induced by particles under rotating magnetic fields. Electric fields can power analogous motility via the electrohydrodynamics of Quincke rotation. This phenomenon occurs when a weakly conducting dielectric particle is suspended in a dielectric liquid of higher conductivity and exposed to a dc electric field. In such conditions, the surface charge of the particle rearranges to form a dipole antiparallel to the applied electric field. Such polarization is highly unstable, and small perturbations of the particle give rise to a mechanical torque that results in steady rotation. Zhang et al. recently demonstrated that the motion of Quincke rollers is also highly dependent on the characteristics of the field.^103^ They employed spherical polystyrene microspheres immersed in a mixture of sodium dioctyl sulfosuccinate (AOT) and hexadecane, sandwiched between two planar electrodes. Below a certain critical field strength, the particles rolled linearly while, above that field strength, they display oscillatory dynamics (Figure 8b).

Applications of self-propelling active colloids aim to exploit two main advantages offered. First, they allow a programming of reliable complex trajectories that enhance motility and facilitate motion in complex environments.^104^ This is known for flagellated microorganisms undergoing 3D motion to scan the environment and identify the optimum direction for chemotaxis. Lee et al. showed that the helically propelling colloids in ac electric fields can tunnel through a porous membrane more efficiently than through linear trajectories (Figure 8c).^11^ This is explained by an increase in the sampling area attributed to the rotational component of helices, which is absent in linear motion. The second advantage offered by active propulsion is that it is not as limited by the external field setup as phoretically driven motion; i.e., active particles have autonomy and programmability in the direction of their swimming. This is particularly relevant in promising biomedical applications such as drug delivery and noninvasive microsurgeries. For example, Schwarz et al. reported the successful intrafallopian transfer of a zygote cell using a spiral-shaped magnetic particle. They demonstrated how a rotating magnetic field can power a microrobotic medical device that collects, transports, and releases microcargo while navigating a cell culture medium (Figure 8d).^105^

## Interplay of Assembly and Propulsion

**Figure 9:**
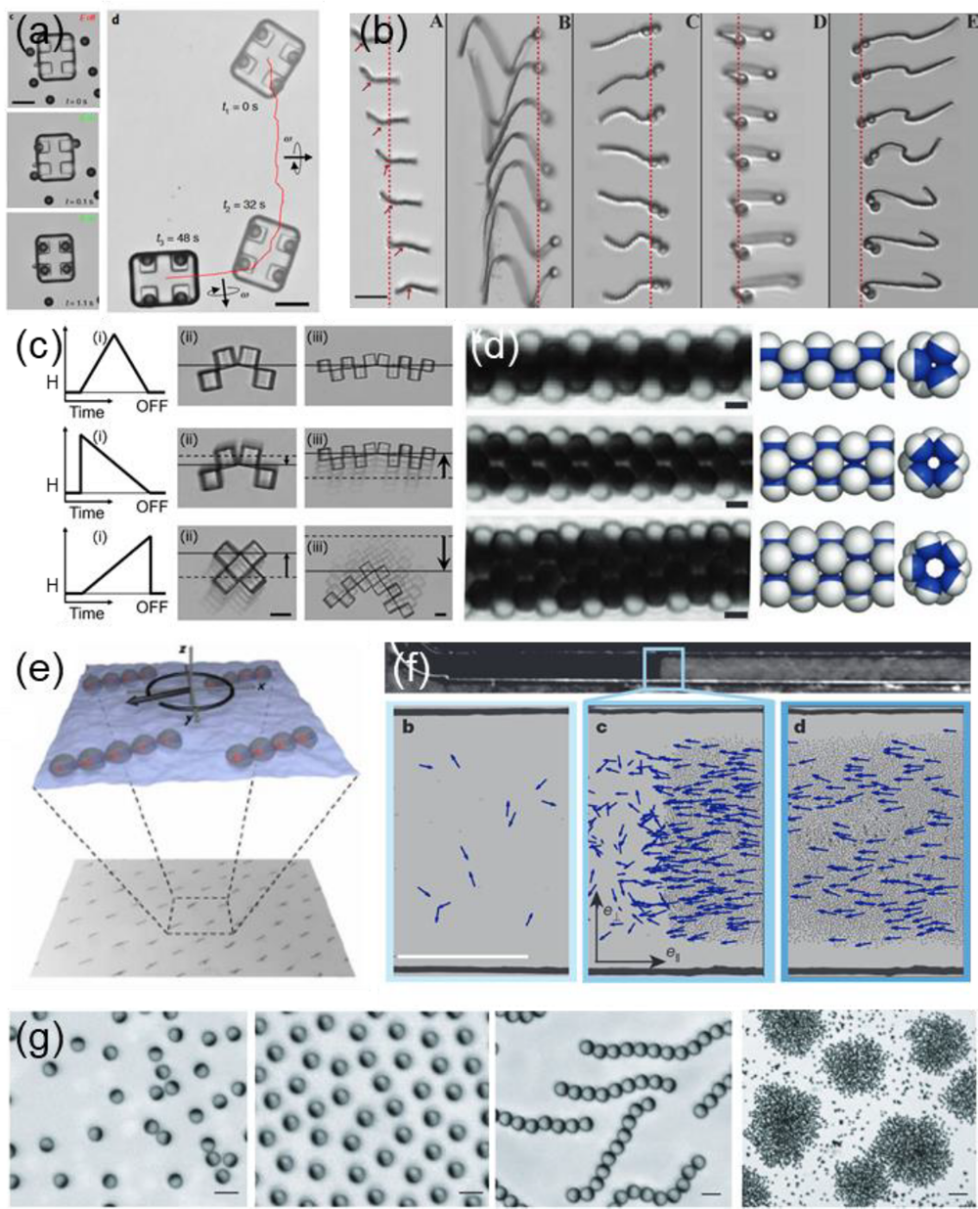
(a) An out-of-plane ac electric field drives the assembly of magnetic microparticles into the wheel pockets of a “microcar” (left panels). A subsequent application of a rotating magnetic field propels the structure (right panel). Scale bars: 25 μm. Adapted with permission from ref (106). Copyright 2020 American Association for the Advancement of Science. (b) Chains assembled under a uniform magnetic field composed of a series of micron-sized superparamagnetic particles. The chains end with larger microparticle “heads” which break the symmetry of motion and allow various 3D motions. Scale bar: 10 μm. Adapted with permission from ref (107). Copyright 2020 National Academy of Sciences. (c) Magnetically assembled “microscallop” device propelled in a shear-thinning fluid by preprogramming the folding and unfolding rates. Scale bar: 10 μm. Adapted with permission from ref (110). Copyright 2020 American Chemical Society. (d) Synchronized tubular microstructures obtained from rotating Janus particles in a precessing magnetic field. Scale bar: 3 μm. Adapted with permission from ref (116). Copyright 2012 Springer Nature. (e) An active spinner phase formed by chains of ferromagnetic microparticles at the water surface. An oscillating magnetic field applied in the same plane causes the spinning of the chains. Adapted with permission from ref (117). Copyright 2020 American Association for the Advancement of Science. (f) A dense swarm of Quincke rollers spontaneously form a propagating band. The colloids are 2.4 μm poly(methyl methacrylate) microspheres in an AOT-hexadecane solution. Scale bar: 500 μm. Adapted with permission from ref (118). Copyright 2013 Springer Nature. (g) ICEP-propelled Janus particles assembled into different states based on the frequency of the external electric field. From left to right, images show a gas phase, swarms, chains, and clusters. Scale bars: 5 μm for the first 3 images, and 30 μm for the last. Adapted with permission from ref (119). Copyright 2016 Springer Nature.

The topics of colloidal assembly and propulsion are vast and multivariate and are often treated separately. This is both for the sake of reducing complexity and because of an implicit understanding that assembly is traditionally induced by static interactions and self-propulsion by dynamic ones. Dissipative assembly and motility are in fact intrinsically related, particularly when powered by the same external energies. Their coupling expands the possibilities for functional applications and gives rise to new highly nonequilibrium phenomena. This includes the propulsion of multicomponent micromachines made of a passive “vehicle” and motile “wheels”. Alapan et al.^106^ demonstrated this principle by fabricating microstructures that are shaped to house spherical magnetic particles. The core microstructure and the magnetic particles are assembled using an electric field, with the final morphology encoded by the shape of the vehicle. Subsequently, the application of a rotating magnetic field induces the particle to rotate and propel the entire machine (Figure 9a). This is one clear example of the hidden potential in combining assembly and propulsion to power dynamic processes using external fields. If the magnetic field is turned off, the machine stops moving. Also, if the electric field is turned off, the machine disassembles. Both effects of the fields are reversible and thus collaborate to give control of multiple functions to search for cargo, incorporate it, and release it at command. Among the most interesting results obtained are those that demonstrate the deep intrinsic relationship between assembly and propulsion, in which one occurs because of the other. Namely, instances of assembly-driven propulsion and propulsion-driven assembly classify two fundamentally new approaches to structure micromaterials and program their movement.

Following the scallop theorem, swimming at a low Reynolds number requires symmetry breaking such that the forces on a swimmer in one direction are not equal in the opposite.^85^ Success in programming the motion of active particles often depends on properly embedding them with anisotropy in shape and surface. A different route employs particles that are fully isotropic but can assemble into microstructures that break the symmetry of the individual components. At that stage, the suprastructure self-propels where the single particle could not. Yang et al. reported the assembly of flexible chains of magnetic microparticles that act as a synthetic flagellum to a larger “head”.^107^ Their spherical superparamagnetic microparticles are assembled into chains using a dc magnetic field after which they are chemically bound by a Michael-addition reaction. These chains have tunable flexibility: they align to a unidirectional magnetic field, bend with a 2D oscillating field, and twist in a 3D precessing field. Propulsion is then introduced to the system by connecting one or more larger particles to either end of a chain. This final assembly step plays the symmetry-breaking role and allows various motions depending on the morphology of the suprastructure (Figure 9b). The same group also demonstrated that such assembly-driven propulsion is also effective in electric fields and evidenced the rotation of asymmetric assembled clusters vs the immobility of chiral ones.^108^ Deriving motility from the geometry of an assembly is an efficient route to functionalizing suprastructures. This is the case for the scallop-like microrobot developed by Han and co-workers.^109−111^ These are structures assembled from polymeric microcubes that are coated on one face with cobalt. Exposed to a uniform and static magnetic field, these particles assemble into chains and remain assembled due to the residual dipole–dipole interaction energy. Toggling the field off and on leads to the bundling and stretching of the chain along the field axis, similar to the opening and closing of a microscopic scallop. In water, such reciprocal motions do not yield any net translation. Conversely, the suprastructure can be engineered to propel in a shear-thinning fluid, where each stroke creates a local viscosity gradient around the microswimmer (Figure 9c).^110^

Propulsion-driven assembly conceptually mirrors the results of assembly-driven propulsion by highlighting the fascinating role of motility in structuring micromaterials.^112−115^ Recent findings have shown that the active motion of colloidal particles contributes to the total pair potential characterizing the interaction between particles. Motility can be viewed as an interaction in addition to all other existing ones, such as dipolar electrostatic, van der Waals, and so on. This implies that activity can potentially improve preexisting assemblies, namely, by increasing the number of interactions, or it can work against attraction and preclude assembly. There are also cases in which assemblies evolve into a unique morphology that only exists in active systems. This can happen because of a synchronization of particle motion due to coupling between interacting particles. Yan et al.^116^ showed how this principle can lead to the assembly of highly complex tubular microstructures composed of Janus colloids in a precessing magnetic field. The particles are silica-based and coated with a thin layer of nickel to induce their magnetic torque-driven oscillation. The rotation of the Janus particles couples with their mutual attraction leading to the eventual synchronization of their motion. This promotes the formation of microtubules where the nickel hemisphere is continuously facing inward (Figure 9d). Synchronization is an emergent phenomenon in propulsion-driven assembly with a novel principle for structuring materials away from equilibrium and endowing them with dynamic properties. Han et al. reported the active assembly of nickel particles at the air–water interface powered by an external rotating magnetic field.^117^ These ferromagnetic microspheres assemble into chains that rotate synchronously with the external magnetic field, forming an active spinner phase (Figure 9e) with the ability to self-heal and tune the motion of passive nonmagnetic particles. Electrically powered active microswimmers and rollers also assemble into nonintuitive dynamic structures. For example, Quincke rollers in dc electric fields spontaneously organize into swarms moving in a single coherent direction.^118^ At low concentrations, the particles assemble into a single flock while, at higher concentrations, they form a polar phase of rollers collectively moving through a confined space (Figure 9f). Such motility-induced phase separation emerges from long-range hydrodynamic interactions that promote collective motion into a macroscopic propagating band. Yan et al. further investigated the structuring of active particles in an electric field, using Janus colloids propelled by ICEP.^119^ Tuning the frequency of the applied ac field, they controlled the asymmetric electrical double layer surrounding the silica and gold hemispheres of the particle. The different ion distribution of the two hemispheres responds differently to the field frequency, leading to the formation of different structures. With an increase in the frequency, the Janus particles reversibly go between gaslike state, swarms, and chains (Figure 9g).

**Figure 10:**
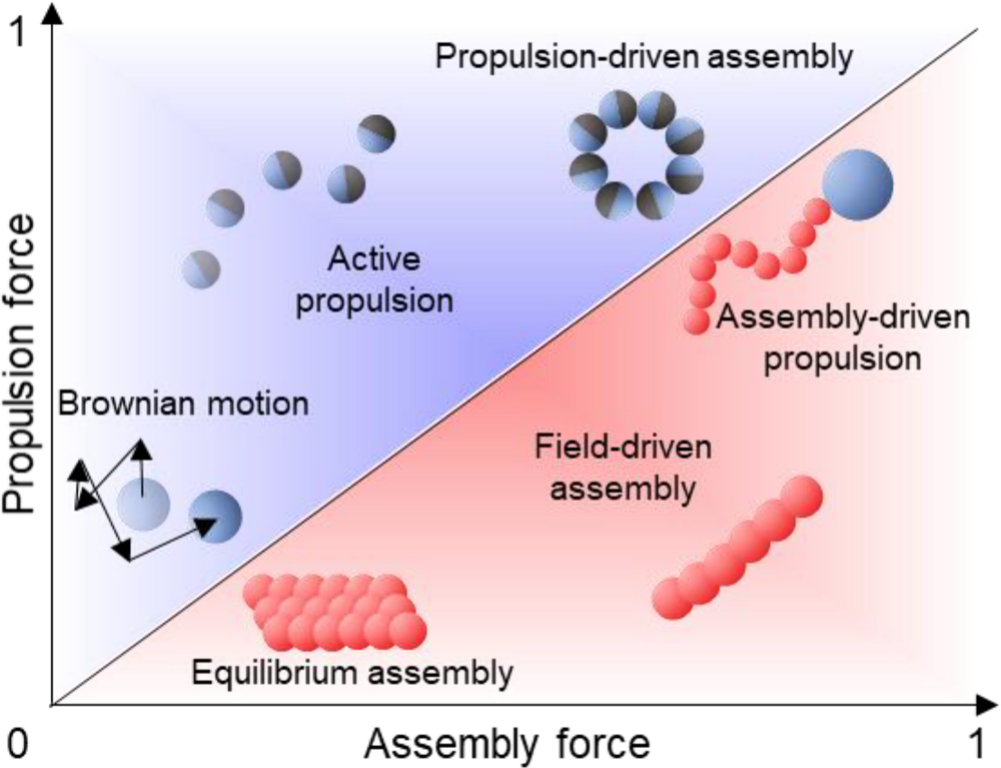
Colloidal assembly and propulsion phenomena are placed in a plot of propulsion force vs assembly force. Mechanisms are classified based on the relative role of potential and kinetic energy in inducing assembly, propulsion, and the host of propulsion-driven assemblies and assembly-driven propulsions.

The traditional difference between assembly and propulsion becomes narrower as more research reveals phenomena that interplay structure and dynamics. The application of an external electric and magnetic field inevitably affects both the morphology of assembly and the kinetics of colloidal motion. Depending on the characteristics of the field, of the particles, and of the medium used, the results can be more dramatically oriented toward an assembled structure or dynamics of motion. However, these two behaviors lie on a spectrum of nonequilibrium phenomena, and a precise definition of said spectrum is far from trivial. Statistical thermodynamics suggests that the proper quantification of nonequilibrium requires a measurement of the entropy production rate. Practically, this is extrapolated from the stochastic fluctuations in a particle system which hide information on the amount of energy dissipation.^120^ From an engineering perspective, it may be useful to approximate the “degree of nonequilibrium” via energy and force balances that reflect the competition of equilibrium potentials and nonequilibrium forces at play.^121^ This would result in dimensionless numbers which, in their most generic form, should be ratios of assembly forces and propulsion forces (or torques) operating on a given colloid. A classification of field-induced phenomena can also be achieved using dimensionless ratios of potential and kinetic energy associated, respectively, with assembly and propulsion. This allows the results from the literature to be placed in a semiquantifiable spectrum based on the assembly and propulsive forces underlying the phenomena, as shown in Figure 10.

## Conclusion

The toolset of electric and magnetic fields provides a large number of design principles to control colloids out-of-equilibrium. These fundamental principles govern static and dynamic assembly as well as passive and active motion. There are many exciting opportunities for techniques on the verge of real-world applications, such as low-cost analytical techniques. In vitro tissue engineering using static magnetic fields is still at the proof-of-concept stage, with the main challenges being the incorporation of magnetic nanoparticles often reducing cell viability and the difficulty in manipulating dense objects. Photonic crystals made with electric fields may become a dye-free and thus more sustainable way to achieve color in many applications.

The strides made in hierarchical assembly have shown much promise as a route to construct microarchitectures, whose functionality will undoubtedly be investigated in upcoming years. Some possibilities we envision are the incorporation of dynamic catalytic machines actuated by magnetic fields within microreactors or the assembly of hybrid materials comprising delicate microscopic parts or cells which would not survive harsh lithographic processing.

Research of self-propelling colloids is at a more fundamental stage compared to assembly, yet synthetic motile microparticles have outstanding potential in drug delivery, bioremediation, and also catalysis. In particular, we expect major advances in controlling motion within complex environments that are more realistic for applications such as in biomedical devices^122−124^ and environmental remediation.^125−127^ These include porous media, suspensions of macromolecules, and cellular environments. Further key research directions are distilled as follows:

(1) understand the role of thermodynamics vs kinetics in the assemblies formed by weak, competing field-induced interactions
(2) deconvolute the respective roles of particle shape, surface chemistry, and dispersing medium in field-induced colloidal phenomena
(3) find new methodologies to override stochastic Brownian forces and achieve organized active motion at the submicron scale by coupling multiple electromagnetic fields
(4) develop new field-induced colloidal platforms that respond to environmental cues and spontaneously self-regulate their structural and temporal characteristics
(5) expand the domain of fundamental active matter research to advanced materials capable of performing sophisticated functions such as energy transfer and mechanical work at the nanoscale

Assembly and propulsion are two of the most fundamental phenomena in nonequilibrium colloid science. One must note that the attempt to bridge living and nonliving matter (as previewed in Figure 1) is not just a route for designing materials but also a unique and truly exciting path for basic discovery. Taking objects that are stochastic by definition and programming their behavior away from equilibrium is an outstanding way to investigate the true meaning of equilibrium itself. The degree to which a system is out-of-equilibrium comes into play strongly yet is still nontrivial to define. Colloids are uniquely suited for identifying and quantifying such concepts, by filling the gap in available experimental models. Straddling the void between living and nonliving, order and disorder, and randomness and determinism is also a way to inquire and hopefully find answers to basic questions on the definition of life and consciousness.^128,129^
